# Differentiation of Acclimation Responses and Seedling Source Effects in Deciduous and Evergreen Conifer Seedlings Under Cross-Altitude Transplantation

**DOI:** 10.3390/plants15182762

**Published:** 2026-09-09

**Authors:** Jiangkai Xie, Zihan Zhang, Yilin Yang, Jinping Guo, Xinjun Chen, Tairui Liu

**Affiliations:** 1 Guangxi Colleges and Universities Key Laboratory for Cultivation and Utilization of Subtropical Forest Plantation, Guangxi Laboratory of Forestry, College of Forestry, Guangxi University, Nanning 530004, China; 18473038789@163.com (J.X.);; 2 Guangxi Forestry Laboratory, Nanning 530004, China; 3 College of Agriculture and Life Sciences, Datong University of Shanxi, Datong 037009, China; 4 College of Forestry, Shanxi Agricultural University, Jinzhong 030801, China; 5 Guangxi Forestry Survey and Design Institute, Nanning 530011, China

**Keywords:** *Larix principis-rupprechtii*, *Picea meyeri*, altitudinal gradient, mountain transplantation, functional traits

## Abstract

Understanding the acclimation responses of conifer species with different functional types in response to altitudinal gradient changes is of significance for deepening our knowledge of how plants cope with global climate change. This study focused on seedlings of larch (*Larix principis-rupprechtii*) and spruce (*Picea meyeri*) within the Pangquangou Nature Reserve in North-central China. By setting two seedling source altitudes (1600 m, 2400 m) and four transplant altitudes (1600 m, 1900 m, 2100 m, 2400 m), we compared the response characteristics of 20 functional traits, including light response, instantaneous photosynthesis, leaf morphology and nutrient storage. The results showed that: (1) Tree functional type was the core factor determining the altitudinal response pattern of seedlings. Larch exhibited higher sensitivity to altitude change, and its photosynthetic (Amax, AQE, LSP, E) and morphological strategy traits (PA, SL, SW, SLA) changed significantly with altitude. (2) The regulation of phenotypic plasticity by seedling source altitude showed a significant functional-type dependence. The larch was significantly regulated by seedling source altitude, with low-altitude seedling sources displaying higher plasticity in traits such as H:D ratio, Tr and NSC. The spruce showed smaller differences between seedling sources, yet exhibited stronger environmental dynamic plasticity than the larch in carbon storage and utilization strategies (Sugars, NSC). (3) The two tree species coped with altitudinal gradient changes through differentiated trait variation pathways. Larch was primarily driven by carbon metabolism and protein synthesis (C, Protein), which was manifested in adjustments of photosynthetic capacity (Pn) and morphology (leaves and branches), thereby achieving rapid adaptation. In spruce, functional traits such as Pn, SLA and NSC were highly coupled; regardless of seedling source, photosynthetic and leaf morphological traits served as the core drivers, and coordinated changes in multiple traits were relied upon to maintain physiological homeostasis.

## 1. Introduction

In the context of global climate change, the functional traits and response strategies of plants determine their environmental adaptability and distribution patterns [1,2,3]. Altitudinal gradients, as integrated environmental factors encompassing temperature, precipitation, light intensity, ultraviolet radiation and soil nutrients, can roughly simulate climatic heterogeneity across thousands of kilometers of latitudinal gradients within a relatively small spatial extent. Mountain altitudinal gradients thus provide a natural experimental platform for studying plant adaptive response mechanisms [4]. Studies have shown that ultraviolet radiation intensifies significantly with increasing altitude, whereas temperature and soil nutrient availability decline, together constituting a comprehensive set of external conditions that shape plant growth and survival strategies [5,6]. Coniferous forests represent a major vegetation type, performing critical ecological functions such as carbon sequestration, soil and water conservation, and biodiversity maintenance. Their response mechanisms to altitudinal gradient changes serve as an important basis for predicting vegetation pattern trends in the mountains of North China under global climate change [7]. Because drastic environmental changes occur across altitudinal gradients, plants often maintain survival by altering the expression of key traits; however, photosynthetic, morphological and leaf nutrient traits do not change synchronously in response to environmental stress. This asynchrony is expected because photosynthetic physiological traits can respond rapidly (within hours to days) to environmental fluctuations, whereas morphological adjustments require longer developmental windows, and leaf nutrient concentrations reflect more integrated, longer-term patterns of nutrient uptake and allocation. Such temporal mismatches imply that species may adopt distinct trait compensation or trade-off strategies under sustained environmental variation [8,9]. Accordingly, we proposed Hypothesis (1): tree species would show species-specific patterns of trait variation when transplanted across altitudes, and photosynthetic, morphological and leaf nutrient traits would show significant differences in their sensitivity to environmental gradients.

Plant functional traits reveal the intrinsic linkages between individual physiological performance and community ecological functions, and can intuitively reflect plant phenotypic responses to environmental changes [10,11]. The Leaf Economics Spectrum (LES) theory has unveiled the global-scale coordination patterns among plant traits, ranging from a “resource-conservative” to a “resource-acquisitive” continuum, in which key functional traits such as specific leaf area, leaf nitrogen content, photosynthetic rate and leaf lifespan exhibit trade-off relationships [12]. Based on this theoretical framework, evergreen conifer species tend to adopt a resource-conservative strategy, characterized by lower SLA, higher leaf dry matter content and longer leaf lifespan, thereby reducing the construction cost per unit leaf area and enhancing resource-use efficiency [13]. In contrast, deciduous conifer species lean towards a resource-acquisitive strategy, displaying higher photosynthetic rates and carbon assimilation capacity [14,15]. Given that resource-acquisitive strategies are typically associated with greater phenotypic plasticity, whereas resource-conservative strategies tend to confer trait stability, the magnitude of plasticity is expected to differ systematically between these functional types. Phenotypic plasticity is thus a crucial capacity for coping with environmental heterogeneity and a key mechanism enabling altitudinal adaptation; yet whether different functional types of plants differ in their dominant traits and plasticity magnitude remains a central question in plant ecology [2]. Considering that phenotypic plasticity serves as the core mechanism for altitudinal adaptation and that its strength is often co-regulated by the species’ genetic background and the environment [16], we proposed Hypothesis (2): the ability of plants to adapt to transplant environments and the magnitude of their plasticity exhibit functional-type dependence, and seedling source altitude modifies the adaptive capacity of tree species.

Two dominant conifer species occur in the mountains of north-central China, where they are concentrated in the cold-temperate coniferous forest belt above 1600 m. The natural distributions of these two species overlap extensively, and they often form mixed communities, together comprising the main body of the montane forest ecosystem in north-central China [17,18]. These two species contrast sharply in terms of leaf functional type. Spruce is an evergreen conifer with perennial leaves, strong low-temperature tolerance and a relatively stable carbon assimilation structure, exhibiting a conservative resource-use strategy in high-altitude habitats [19,20]. Larch, by contrast, is a deciduous conifer that reduces damage from extreme low temperatures and frost through winter leaf shedding and rapidly accumulates biomass during the short growing season, leaning toward a resource-acquisitive adaptive strategy [21]. For individual species, existing studies have shown that larch seedlings adjust their photosynthetic parameters, leaf morphology and carbon-nitrogen metabolic traits adaptively when transplanted across altitudes. High-altitude seedling sources primarily stabilize function by optimizing photosynthetic structures, whereas low-altitude seedling sources mainly rely on soluble proteins and leaf area adjustments, exhibiting stronger physiological plasticity [22,23]. In spruce, high-altitude seedling sources influence physiological functions mainly through nutrient changes, while low-altitude seedling sources depend more on indirect regulation of nutrient and photosynthetic functions via morphological traits [24]. However, comparative studies of these two species under the same transplant altitudinal gradients are still lacking. The differences in trait response magnitude between the evergreen and deciduous functional types, the way in which seedling source altitude regulates the functional trait plasticity of the two species, and the correlation characteristics among traits have not yet been clearly elucidated empirically. These contrasting resource-use strategies suggest that the two species rely on fundamentally different drivers of trait coordination. Therefore, we proposed Hypothesis (3): larch tends to drive physiological responses through flexible adjustments of morphological structures, while spruce relies on tight coordination among photosynthetic, growth and nutrient traits to cope with environmental change. This study compared the functional trait responses of spruce and larch along altitudinal gradients to elucidate the environmental adaptation strategies and regulatory mechanisms of conifer species with contrasting functional types.

## 2. Research Methods

### 2.1. Study Area

This study was conducted in the Pangquangou National Nature Reserve on Guandi Mountain, Jiaocheng County, Lüliang City, Shanxi Province (37°45′–37°55′ N, 111°22′–111°33′ E), at the junction of Jiaocheng, Fangshan and Loufan counties. The reserve extends 15 km from north to south and 14.5 km from east to west, covering a total area of 104.435 km^2^. The vegetation within the reserve is well preserved, with a forest coverage rate as high as 85%; the forested area amounts to 77.097 km^2^, the shrubland area covers 11.659 km^2^, and the growing stock volume of living trees reaches 1.3 million m^3^. The forest types consist predominantly of pure natural secondary forests of larch (*Larix principis-rupprechtii*), spruce (*Picea meyeri*) and Chinese pine (*Pinus tabuliformis*), together with a small proportion of plantations. The reserve belongs to the warm-temperate semi-humid climatic zone and represents a climatic transition belt from the mid-temperate to the warm-temperate zone, characterized by a mean annual temperature of 4.3 °C, a relative humidity of 70%, and a mean annual precipitation of approximately 820 mm. A pronounced altitudinal gradient spans 1331 m (from 1500 m to 2831 m), forming a distinct vertical vegetation spectrum. The vegetation is mainly composed of cold-temperate coniferous forest, cold-temperate mixed coniferous and broad-leaved forest, and temperate broad-leaved mixed forest. Based on the dominant species, it can be broadly divided into five types: pure natural secondary larch forest, spruce forest, Chinese pine forest, poplar-birch broad-leaved forest, and Liaodong oak (*Quercus wutaishanica*) forest.

### 2.2. Experimental Design and Plot Selection

The experimental site was located in the Chailugou gully within the Xiaowenshan Forest Farm. At both 1600 m and 2400 m elevations, forests of spruce and larch are present. From each elevation, 300 spruce seedlings were selected from the spruce forest and 300 larch seedlings from the larch forest, giving a total of 600 spruce seedlings and 600 larch seedlings (all 2–3 years old, 50 ± 10 cm in height). Seedlings collected at 1600 m were designated as the low-altitude source, and those collected at 2400 m as the high-altitude source. Four 20 m × 20 m plots were established along the altitudinal gradient and labeled, from low to high altitude, as AL-1600, AL-1900, AL-2100 and AL-2400. The seedlings were lifted in late September 2018 and transplanted immediately thereafter. The spruce and larch seedlings were each randomly divided into four groups. Following a split-plot experimental design, the four groups of the two species were transplanted to four habitats at altitudes of (1600 ± 50) m, (1900 ± 50) m, (2100 ± 50) m and (2400 ± 50) m. At each habitat, three forest gaps of 20 m × 20 m were established as replicates, with a minimum spacing of 30–50 m between adjacent gaps to ensure their independence. Within each gap, five seedlings per species × source altitude combination were randomly selected for trait measurements, and their values were averaged to yield a single gap-level mean for each trait. The gaps were selected with uniform distribution, moderate spacing and similar light conditions to ensure consistency of environmental factors. In all statistical analyses, the three forest gaps at each altitude were treated as experimental replicates (*n* = 3), thereby avoiding pseudoreplication at the individual seedling level.

### 2.3. Measurement of Physiological Characteristics

#### 2.3.1. Measurement of Light Response Parameters and Instantaneous Photosynthetic Parameters

Photosynthetic measurements were conducted during the peak growing season in late July 2020, on clear, windless mornings (09:00–11:30). In each plot, for each species and each seedling source altitude, five healthy, pest-free seedlings were randomly selected. A LI-6400 and a LI-6800 portable photosynthesis system (LI-COR Inc., Lincoln, NE, USA) were used to measure the seedlings of the two conifer species. To obtain a suitable and stable environment, a CO_2_ cylinder was used to provide a stable CO_2_ source of 380 ppm during determination. During all measurements, the leaf chamber temperature was controlled at 25 °C [25,26], and the flow rate was adjusted to maintain the leaf-to-air vapor pressure deficit (VPD) within 1.2–1.8 kPa. The built-in light source was employed to set a photosynthetic active radiation (PAR) gradient sequence to generate light-response curves. For both species, the saturated light intensity was simulated at 1200 μmol m^−2^ s^−1^, and the PAR gradient was set as 0, 50, 100, 200, 400, 600, 800, 1000, 1200, 1400 and 1600 μmol m^−2^ s^−1^. Net photosynthetic rate (Pn) and transpiration rate (E) were measured under a range of photosynthetically PAR levels to construct light-response curves. The light-response curves were then fitted using the non-rectangular hyperbola model to determine the maximum net photosynthetic rate (Amax), light saturation point (LSP), and light compensation point (LCP) [27,28]. Linear regression was performed on the low-light region of the light-response curve, and the slope was taken as the apparent quantum efficiency (AQE).

(1) $$Pn=\frac{\alpha I+Amax-\sqrt{{(\alpha I+Amax)}^{2}-4\theta \alpha IAmax}}{2\theta }-Rd$$

#### 2.3.2. Measurement of Morphological Parameters

In August 2020, morphological parameters of the two conifer seedlings in the four transplant plots were measured. In each plot, for each species and each seedling source altitude, five healthy, pest-free seedlings were randomly selected. A steel ruler and a digital vernier caliper were used to measure current-year stem length (BL) and basal diameter (DBA). Healthy, fully developed sun-exposed needles from the middle-upper part of the seedlings in each treatment were collected and stored in a low-temperature insulated box. Fresh needle samples were cleaned and laid flat on a scanner, and high-resolution images were acquired. Leaf length (SL) and leaf width (SW) were obtained from these images, and the leaf length-to-width ratio was calculated as LW = SL/SW. After measurement, the needles enclosed in the cuvette were excised and scanned, and the projected leaf area (PA) was determined from the scanned images using the WinSEEDLE leaf analysis system [29]. The same needles were then oven-dried at 65 °C for 48 h to constant weight, and the dry weight (DW) was measured using an electronic balance. The specific leaf area (SLA = PA/DW) was then calculated.

#### 2.3.3. Measurement of Nutrient and Carbon Allocation Parameters

In each plot, healthy leaves were randomly collected from five high-altitude and five low-altitude seedlings of each species, mixed thoroughly, and then divided into three subsamples. For both species, soluble sugar content (Sugars) and starch content (Starch) were determined by the anthrone colorimetric method, and non-structural carbohydrate content (NSC = Sugars + Starch) was calculated. For spruce seedlings, soluble protein content (Protein) was measured by UV absorption; total carbon content (C) was determined with a total carbon analyzer; and leaf total nitrogen content (N) was measured by the Kjeldahl method. For larch seedlings, soluble protein content was determined using a standard colorimetric method, and leaf total carbon (C) and total nitrogen (N) contents were measured with an elemental analyzer (Vario EL III, Elementar Analysensysteme GmbH, Langenselbold, Germany).

### 2.4. Data Analysis

Statistical analyses were performed using R version 4.5.3. Multi-factor ANOVA was applied to evaluate the effects of tree species, seedling source altitude, transplant altitude and their interactions on each functional trait. For indicators with significant differences (*p* < 0.05), Tukey’s HSD test was used for multiple comparisons, and the ggplot2 package was employed to generate bar charts with significance letters to visualize trend differences among treatments. The phenotypic plasticity index (PI) was calculated to assess the sensitivity of each trait to environmental change, using the formula PI = (Xmax − Xmin)/Xmax; this index ranges from 0 to 1, with higher values indicating greater plasticity. Here, Xmax and Xmin refer to the maximum and minimum mean trait values, respectively, among the different transplant altitude treatments for each species and seedling source combination. Pearson correlation analysis was conducted using the pheatmap package to examine the intrinsic associations among functional traits. Finally, redundancy analysis (RDA) was performed on the functional traits of the two tree species to clarify the explanatory power of transplant altitude variation on the functional trait variation of larch and spruce under different seedling source altitudes, as well as the relative contributions of each trait.

## 3. Results

### 3.1. Multi-Factor ANOVA of Species, Seedling Source Altitude and Transplant Altitude Effects

We analyzed the significance of the main effects of species, seedling source elevation, and transplanting elevation, as well as their interactions on traits. As shown in Table 1, species had an significant effect on 18 traits (*p* < 0.05), with no significant effect only on AQE and NSC. Seedling source elevation had an extremely significant effect on light-response and instantaneous photosynthetic parameters (Amax, LCP, AQE, Pn, WUE), morphological parameters (SL, SW, PA, BL, H:D ratio), and the nutrient parameter (N). It significantly affected morphological index parameters (SLA, H:D ratio) and the nutrient parameter (Protein), as well as LSP among the light-response parameters. The transplanting elevation effect significantly affected instantaneous photosynthetic parameters (Pn, WUE), morphological parameters (SL, PA, LWRatio), and nutrient parameters (Sugars, C, N). It also significantly affected SLA among morphological indices, as well as LCP in the light-response parameters, H:D ratio in the morphological parameters, and Protein in the nutrient parameters. The three-way interaction among species, seedling source elevation, and transplanting elevation significantly affected the light-response indicator Amax, morphological parameters (LWRatio, BL), and nutrient indicators (Protein, Sugars). It also significantly affected AQE and Pn among the light-response and instantaneous photosynthetic parameters, as well as instantaneous photosynthetic parameters (E, WUE) and NSC in the morphological parameters.

### 3.2. Differences in Functional Traits Between Tree Species Under Different Altitude Treatments

#### 3.2.1. Differences in Light-Response Parameters Between Tree Species Under Different Altitude Treatments

For the low-altitude seedling source (1600 m) of larch, the light-response parameters (Amax, LSP, LCP) first increased and then decreased with increasing transplant altitude, whereas spruce showed no pronounced changes. The AQE of the low-altitude seedling sources of both species first decreased and then increased with altitude. For the high-altitude seedling source (2400 m) of larch, Amax, AQE and LSP peaked at mid-to-high altitudes, while LCP did not vary markedly with altitude. For the high-altitude seedling source of spruce, Amax, AQE and LCP generally increased with altitude.

**Figure 1:**
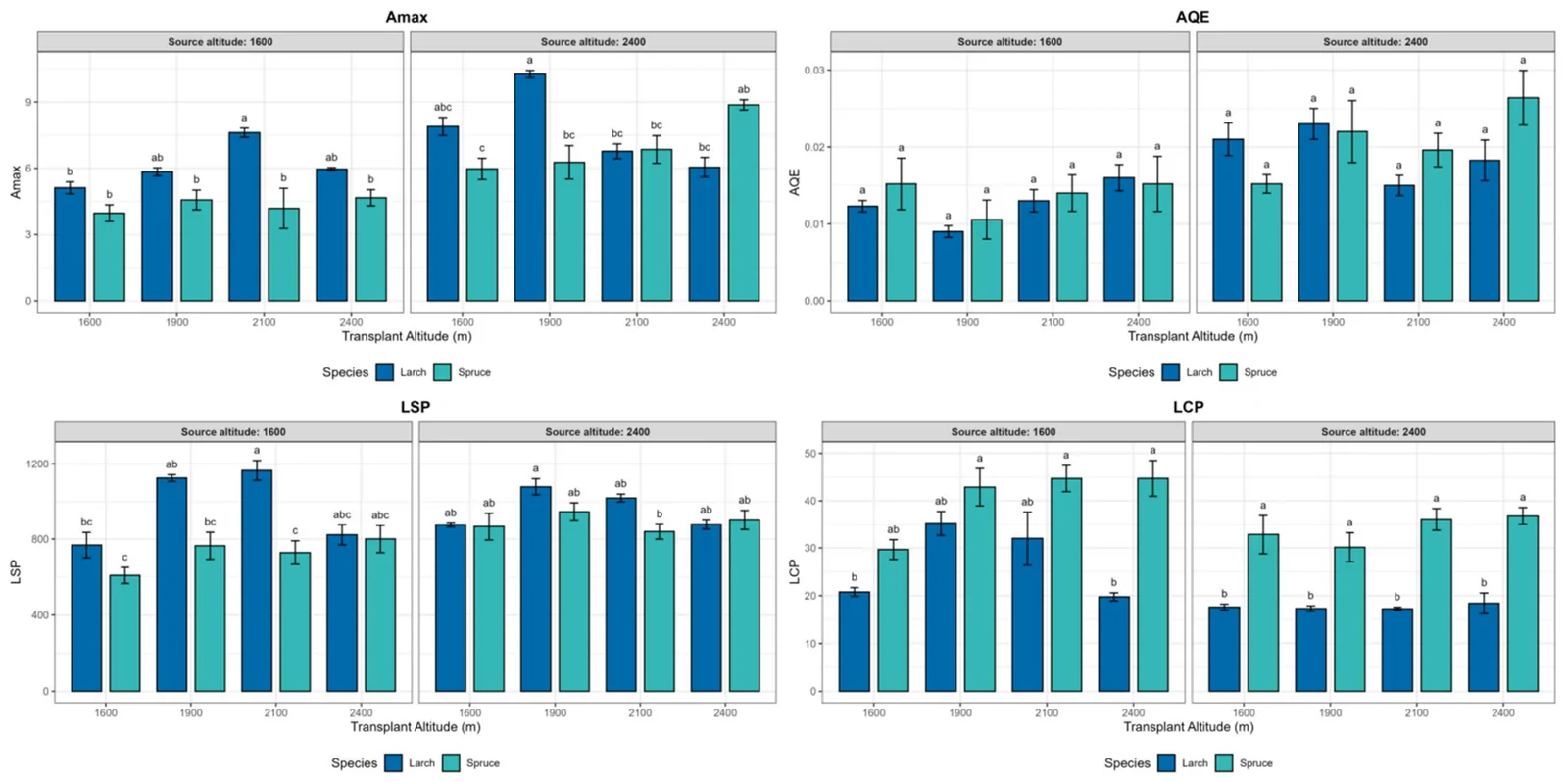
Amax (μmol CO_2_ m^−2^ s^−1^), AQE (mol CO_2_ mol^−1^ photons), LSP (μmol photons m^−2^ s^−1^), LCP (μmol photons m^−2^ s^−1^), *n* = 5. Error bars represent ±SE and different lowercase letters indicate significant differences based on Tukey’s HSD post-hoc tests (*p* < 0.05).

The comparison revealed that the light-response characteristics of larch responded more strongly to the altitudinal gradient, with pronounced differences among transplant altitudes (Figure 1).

#### 3.2.2. Differences in Instantaneous Photosynthetic Parameters Between Tree Species Under Different Altitude Treatments

For the low-altitude seedling source (1600 m) of larch, the instantaneous photosynthetic parameters (Pn, WUE) first decreased and then increased with increasing altitude, while E showed the opposite pattern. For the low-altitude seedling source of spruce, E and Pn first increased and then decreased with altitude, whereas WUE showed no pronounced change. The high-altitude seedling source (2400 m) of larch exhibited a smaller overall variation with transplant altitude compared to its low-altitude source, while the instantaneous photosynthetic parameters (E, Pn, WUE) of the high-altitude spruce source increased slightly with altitude.

**Figure 2:**
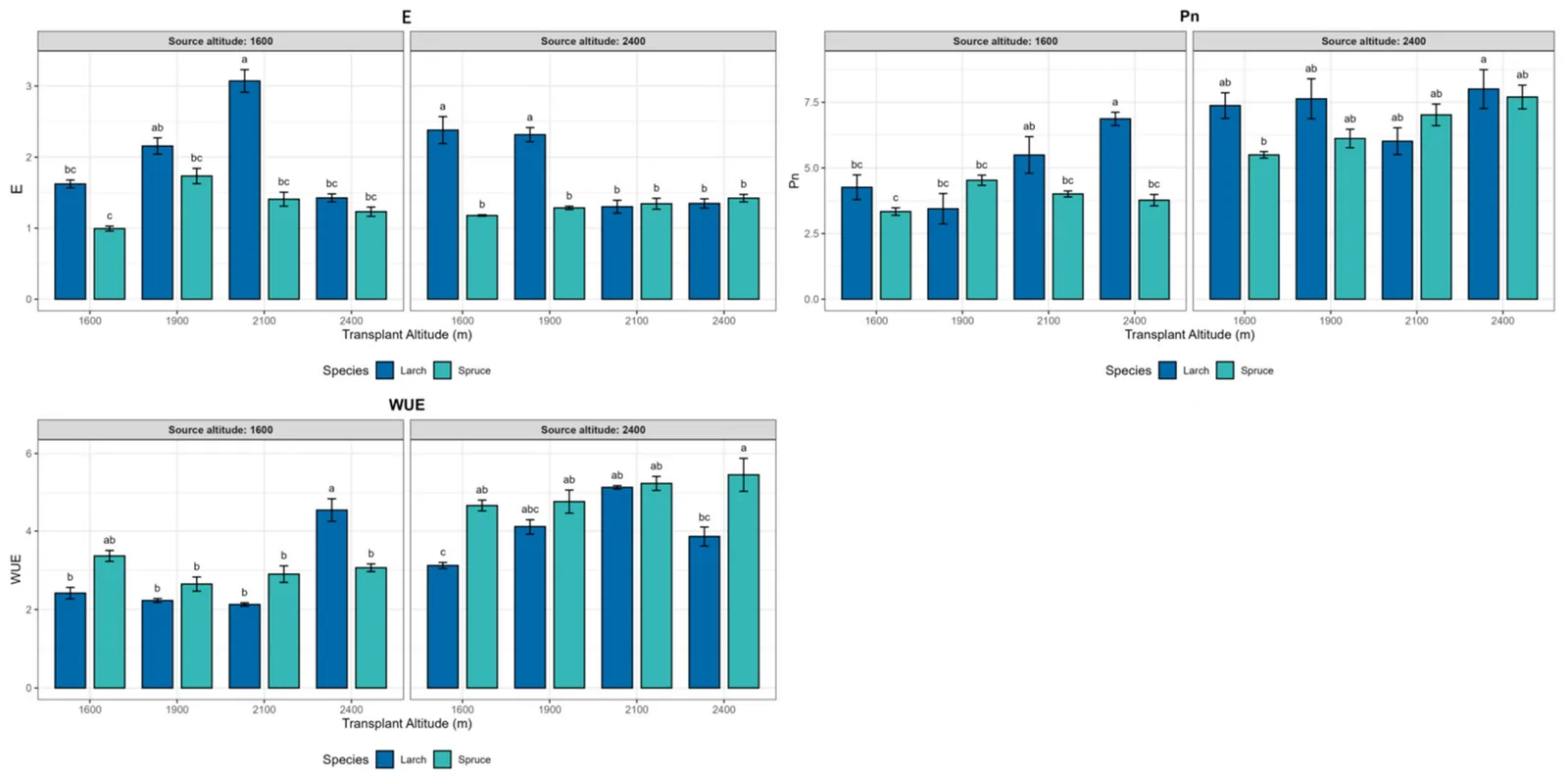
E (mmol H_2_O m^−2^ s^−1^), Pn (μmol CO_2_ m^−2^ s^−1^), WUE (μmol CO_2_ mmol^−1^ H_2_O), *n* = 5. Error bars represent ±SE and that different lowercase letters indicate significant differences based on Tukey’s HSD post-hoc tests (*p* < 0.05).

The comparison indicated that the changes in Pn and E across transplant altitudes were generally greater in larch than in spruce (Figure 2).

#### 3.2.3. Differences in Morphological Parameters Between Tree Species Under Different Altitude Treatments

For the low-altitude seedling source of larch, the morphological parameters (PA, SL, SW, H:D ratio) first increased and then decreased with altitude; SLA and LWRatio showed the opposite pattern, and BL and DBA declined continuously with increasing altitude. For the low-altitude seedling source of spruce, the morphological parameters (PA, SL, SW, SLA) first increased and then decreased with altitude, peaking at mid-altitudes, while LWRatio, BL, DBA and H:D ratio increased slightly and continuously with altitude. For the high-altitude seedling source of larch, the morphological parameters (PA, SL, SW, LWRatio, H:D ratio) rose continuously with altitude, whereas the other indicators showed no marked changes. For the high-altitude seedling source of spruce, the morphological parameters (PA, SL, SW, SLA, LWRatio, BL, DBA) increased slightly and continuously with altitude, while H:D ratio showed the opposite pattern.

**Figure 3:**
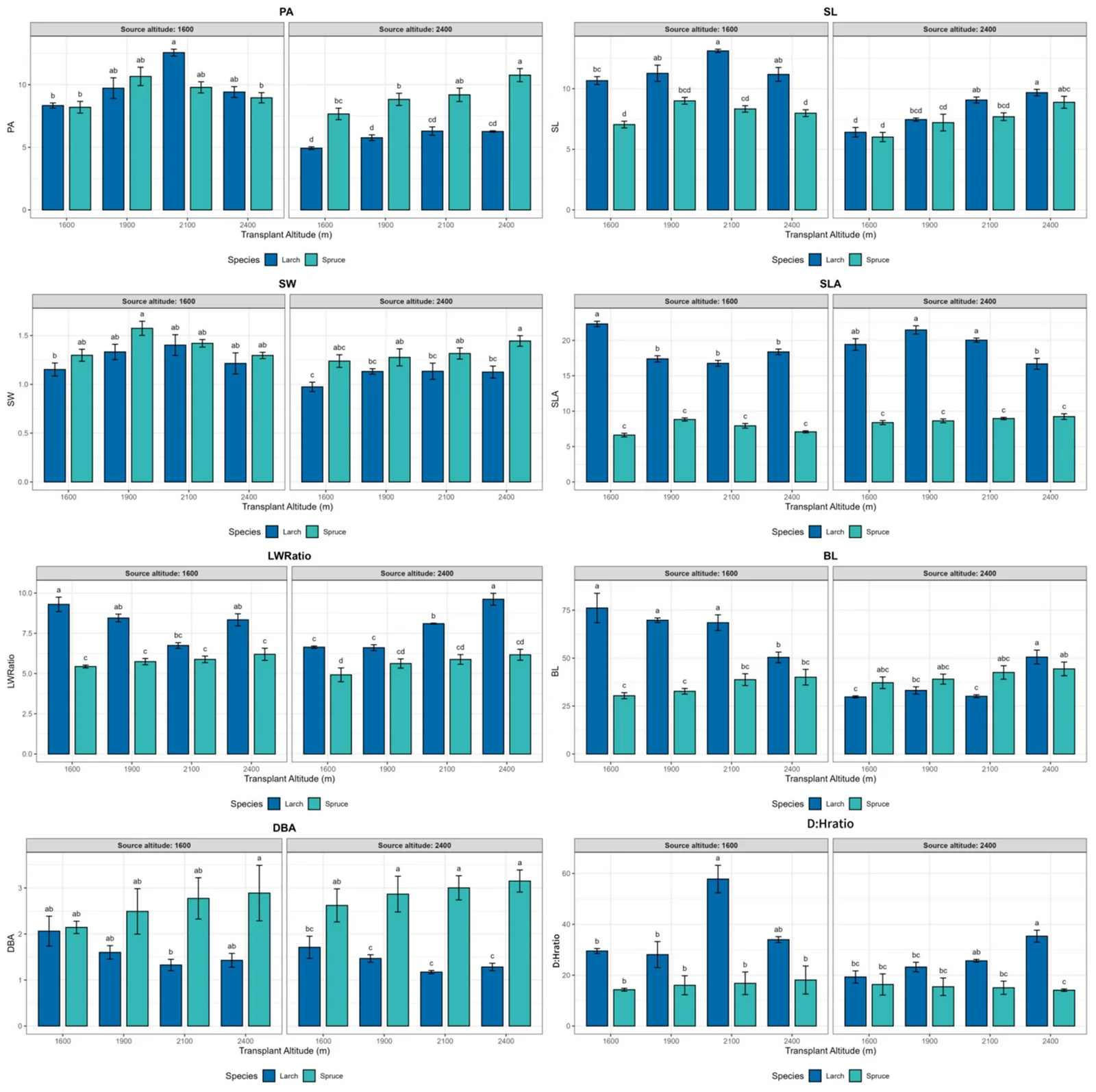
SL (cm), SW (cm), PA (cm^2^), SLA (cm^2^ g^−1^), BL (cm), DBA (mm), *n* = 5. Error bars represent ±SE and different lowercase letters indicate significant differences based on Tukey’s HSD post-hoc tests (*p* < 0.05).

The morphological parameters of larch exhibited greater overall variation across the transplant altitude gradient than those of spruce, indicating higher sensitivity to altitudinal habitat changes, which was consistent with the response patterns observed for light-response and instantaneous photosynthetic parameters (Figure 3).

#### 3.2.4. Differences in Nutrient Parameters Between Tree Species Under Different Altitude Treatments

For the low-altitude seedling source of larch, the nutrient parameters (Protein, C, N) first decreased and then increased with altitude, while Sugars and NSC showed the opposite pattern. For the low-altitude seedling source of spruce, the nutrient parameters (Sugars, NSC, C) first increased and then decreased, whereas Protein and N declined continuously. For the high-altitude seedling source of larch, Protein and N first decreased and then increased with altitude, and Sugars and C rose continuously. For the high-altitude seedling source of spruce, the nutrient parameters (Sugars, NSC, C) increased continuously with altitude, whereas Protein and N first decreased and then increased.

**Figure 4:**
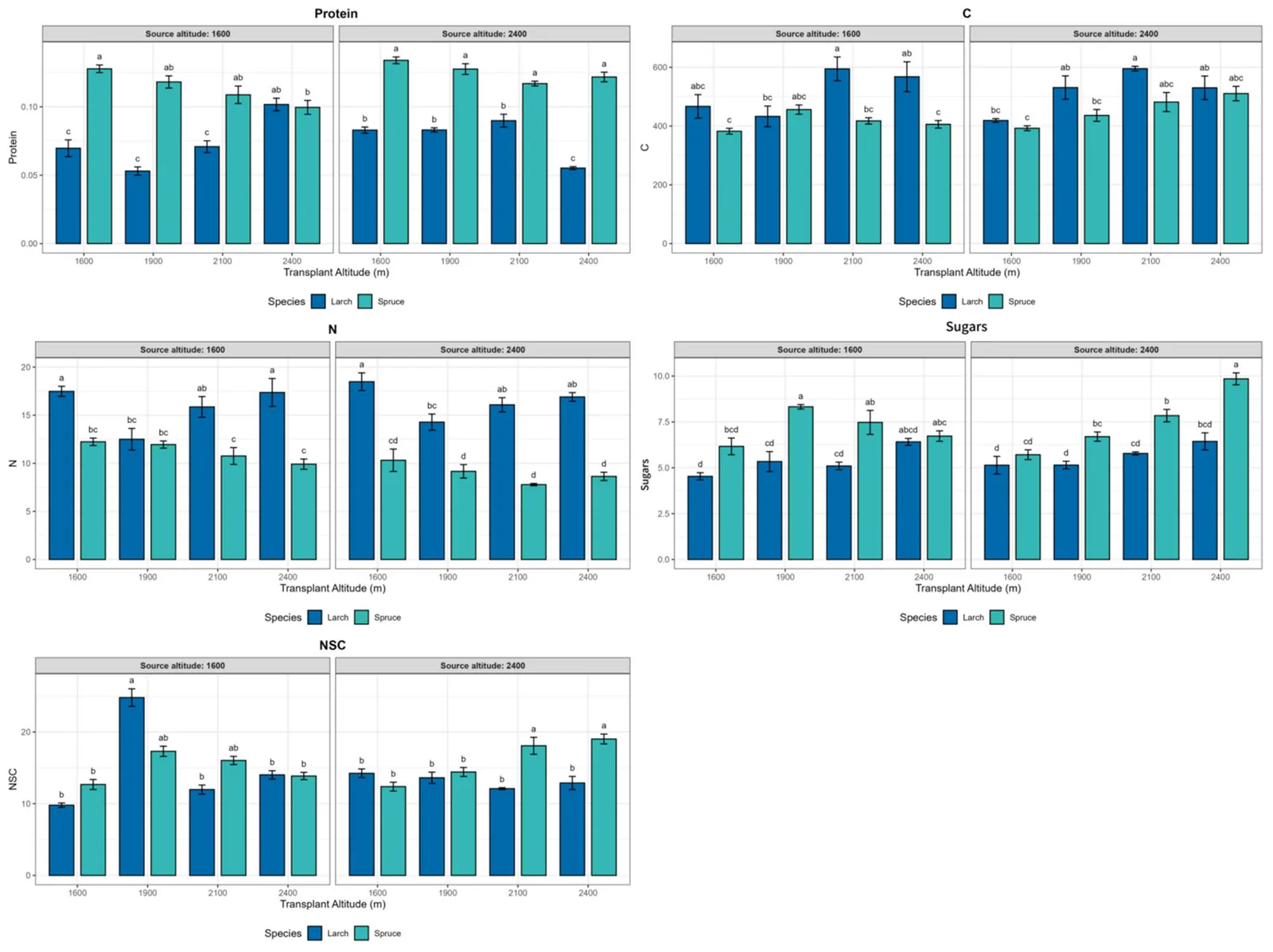
Protein (mg g^−1^), C (mg g^−1^), N (mg g^−1^), Sugars (mg g^−1^), NSC (mg g^−1^), *n* = 5. Error bars represent ±SE and different lowercase letters indicate significant differences based on Tukey’s HSD post-hoc tests (*p* < 0.05).

The comparison revealed that the fluctuations in Protein, NSC and C across transplant altitudes were greater in larch than in spruce, indicating greater altitudinal response differences, while the nutrient and carbon allocation parameters of spruce changed relatively moderately (Figure 4).

### 3.3. Phenotypic Plasticity Analysis of Functional Traits Between Tree Species

In the low-altitude seedling source, the mean PI across the 20 traits for larch was 0.3705, which was higher than that for spruce. Among the light response and instantaneous photosynthetic parameters as well as the nutrient parameters, the mean PI of larch exceeded that of spruce, with particularly large differences observed in WUE, Pn, E, Amax, NSC, and Protein. Among the morphological parameters, H:D ratio, DBA and BL differed considerably, whereas SL, SW and SLA showed no marked differences, displaying a relatively consistent plasticity level.

In the high-altitude seedling source, the mean PI across the 20 traits was 0.2943 for larch and 0.2420 for spruce, and the overall gap between the two species was smaller than that observed in the low-altitude source. Among the light response and instantaneous photosynthetic parameters, the difference in mean PI between the two species narrowed; E and WUE still exhibited higher values in larch than in spruce, whereas AQE, Pn and LCP showed higher PI values in spruce. Among the morphological parameters, the mean PI of larch remained higher than that of spruce, with substantial differences in H:D ratio, BL and DBA. Among the nutrient parameters, the mean PI values of the two species were close; however, larch showed a higher PI for Protein, while spruce displayed higher PI values for Sugars and NSC.

**Figure 5:**
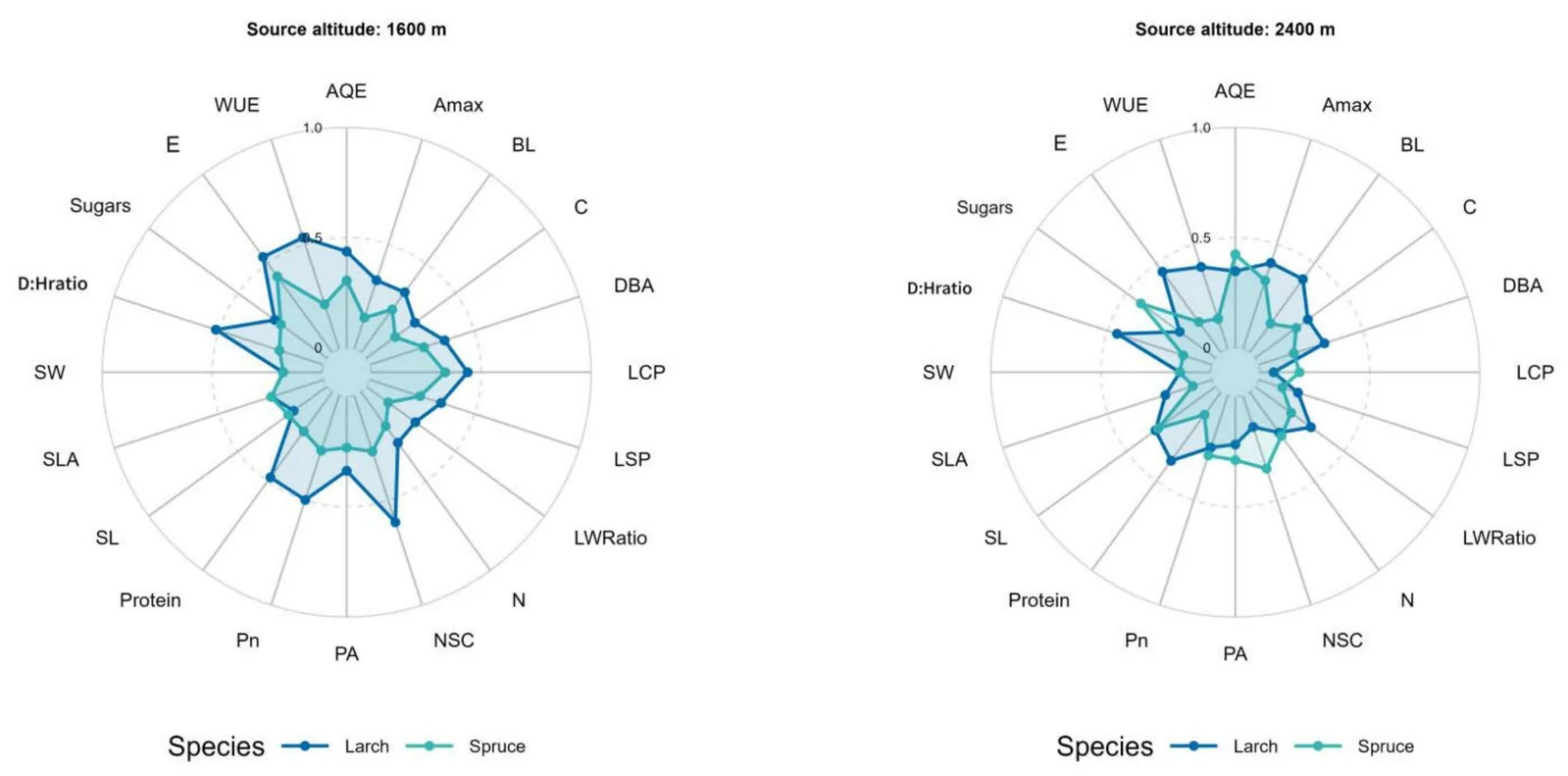
Phenotypic plasticity analysis of 20 traits in seedlings from low-elevation (1600 m) and high-elevation (2400 m) seedling sources.

The comparison revealed that the overall mean PI across the 20 traits of larch was higher than that of spruce under both seedling source conditions. Under the low-altitude seedling source, larch had higher PI values than spruce in 18 out of the 20 traits. Under the high-altitude seedling source, larch showed higher PI values than spruce in 12 traits. For both species, the mean PI under the low-altitude seedling source was higher than that under the high-altitude source. These results indicate that the overall magnitude of response to transplant altitude change was greater in low-altitude seedling sources than in high-altitude ones; larch exhibited higher overall phenotypic plasticity than spruce in response to altitude change; and this difference was more pronounced in the low-altitude seedling source (Figure 5).

### 3.4. Correlation Analysis of Functional Traits Between Tree Species

The functional trait correlations of larch exhibited a pronounced modular pattern, which could be divided into two correlation modules. The first module was dominated by morphological traits, in which H:D ratio, SW, BL, PA and SL were significantly positively correlated with one another. Among them, PA and SL showed the highest correlation coefficient, indicating a high degree of coordinated variation among stem morphological traits. This module was negatively correlated with photosynthetic traits (Amax, AQE, Pn, WUE), with the negative correlations of BL with WUE and of SL with AQE being the most prominent. The second module was primarily composed of light response and instantaneous photosynthetic traits, within which Amax, AQE and Pn were significantly positively correlated; WUE showed positive correlations with Pn and AQE, E was positively correlated with LSP, and E was significantly negatively correlated with LWRatio. Among nutrient traits, NSC was positively correlated with LSP, N was weakly negatively correlated with Amax, Protein displayed a relatively strong positive correlation with WUE, and Sugars was only weakly correlated with C. SLA was generally negatively correlated with morphological traits, with a relatively pronounced negative correlation with H:D ratio.

**Figure 6:**
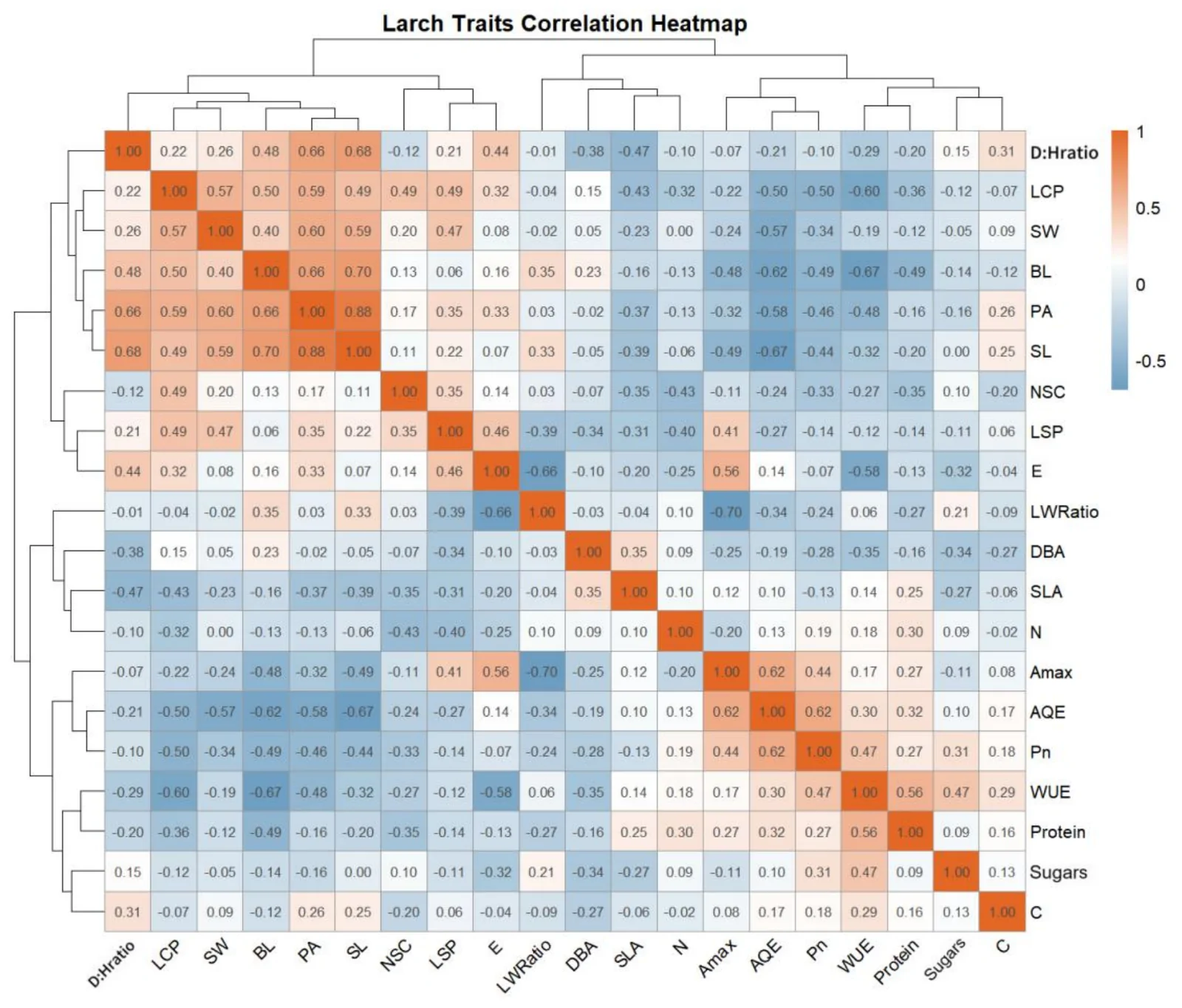

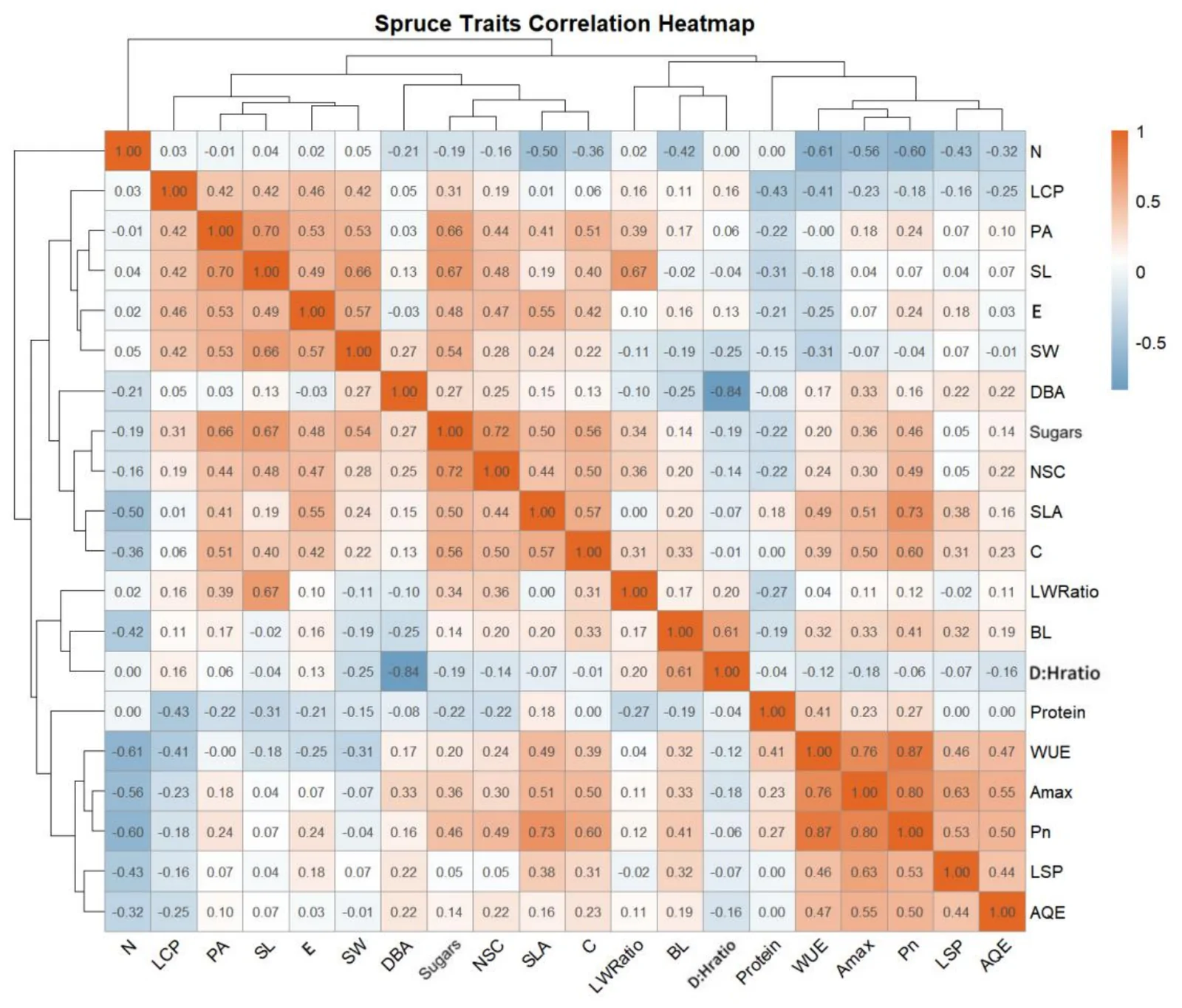
Correlation analysis of 20 functional traits in larch and spruce.

Spruce displayed a tight and stable trait correlation network. The internal coordination among light response and instantaneous photosynthetic traits was stronger, with WUE, Amax, Pn, LSP and AQE all significantly positively correlated with one another. Notably, the correlation coefficients between Amax and Pn, and between Amax and WUE, were both higher than the corresponding coefficients in larch, indicating a greater degree of coordination among light response and instantaneous photosynthetic traits in spruce. Among morphological traits, PA, SL, E and SW were significantly positively correlated; LWRatio was positively correlated with SL and PA, whereas DBA and H:D ratio exhibited a strong negative correlation. Regarding nutrient traits, Sugars was significantly positively correlated with PA, SL and NSC; SLA showed positive correlations with both Pn and E; and N was relatively strongly negatively correlated with photosynthetic traits (WUE, Amax, Pn), a pattern that was not evident in larch. Protein and H:D ratio were weakly correlated with most traits, and their variation patterns differed considerably (Figure 6).

### 3.5. Redundancy Analysis of Functional Traits Between Tree Species

**Figure 7:**
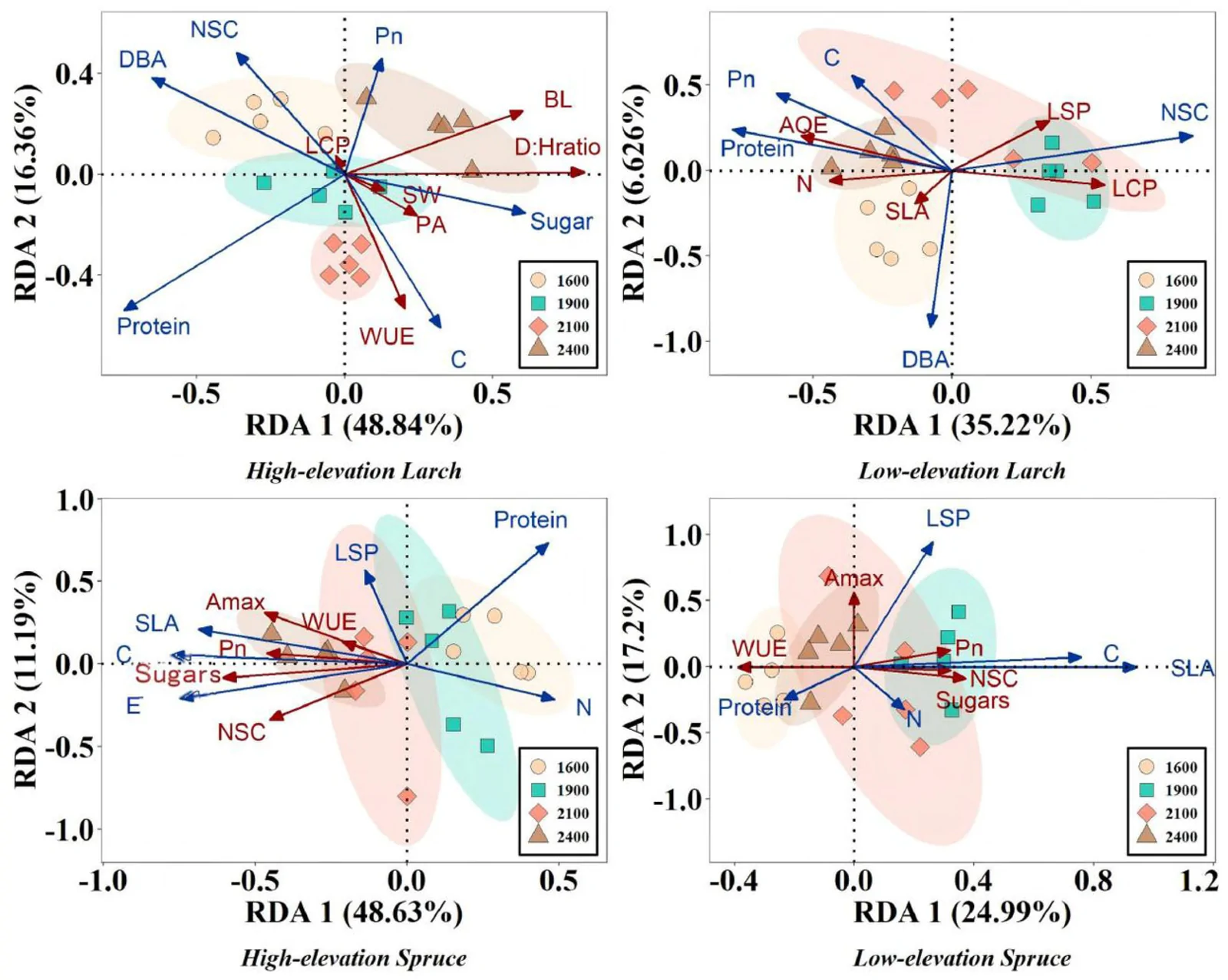
Redundancy analysis of functional traits of larch and spruce from low-elevation (1600 m) and high-elevation (2400 m) seedling sources.

The RDA results revealed a common pattern: the explained variation in functional traits at high-altitude environments (59.82–65.2%) was markedly higher than that at low altitudes (approximately 42%), indicating that the native high-altitude environment exerted a more pronounced influence on the functional trait variation of seedlings. However, the two species adopted fundamentally different physiological trade-off strategies at high altitudes. The core differentiation of larch was reflected in carbon allocation (C, NSC) and protein synthesis (Protein) strategies, whereas spruce exhibited a strong coupling among photosynthetic traits, leaf morphology and nutrient storage, relying more on the enhancement of photosynthetic capacity and nitrogen assimilation to cope with altitudinal change. Overall, the divergence in the main driving factors between the two species demonstrates that they respond to the altitudinal gradient through distinct strategic differentiation (Figure 7).

## 4. Discussion

### 4.1. Differences in Functional Trait Responses of Tree Species to Cross-Altitude Transplantation

The results of multi-factor ANOVA showed that tree species was the most important driver of functional trait differentiation between the two species in the analyses, which is consistent with the large differences expected between deciduous and evergreen leaf habits: evergreen and deciduous tree species differ intrinsically in leaf functional type, a difference shaped by long-term natural selection that persists irrespective of changes in transplant altitude. Seedling source altitude had particularly pronounced effects on photosynthetic and morphological traits, indicating that the native environment exerts a lasting influence on seedling functional traits; i.e., seedlings carry physiological and structural information from their place of origin and continue to express it even after the environment changes [30,31]. The transplant altitude effect was mainly manifested in photosynthetic strategies and nutrient storage, suggesting that temperature, light and soil nutrient conditions of the new habitat can rapidly regulate the carbon assimilation and resource allocation strategies of both species, while the magnitude of such responses is constrained by adaptation to the native environment [32,33]. The significant three-way interaction of seedling source × transplant altitude × species indicates that the trait differences between the two species are not caused by a single factor, but result from the combined effects of species differences and altitudinal gradients.

The low-altitude seedling source of larch responded to changes in transplant altitude with a substantially greater magnitude than that of spruce, which is consistent with the typical “resource-acquisitive” strategy of deciduous conifers. In low-altitude regions with longer growing seasons, this strategy enables larch to achieve rapid growth through high investment. However, the regulatory effect of transplant altitude on spruce was rather limited, and it did not create environmental differences sufficient to induce changes in leaf morphological structure; notably, the variation in morphological parameters of the low-altitude source was markedly smaller than that of larch. For the low-altitude seedling sources of both species, light-response parameters (Amax, LSP) and morphological parameters (PA, SL, SW) all exhibited a unimodal pattern of first increasing and then decreasing with increasing transplant altitude, indicating that mid-altitude transplant sites can provide more favorable light and temperature conditions for low-altitude sources, thereby facilitating the construction of photosynthetic structures [34,35]. In terms of nutrient traits, the variation in Protein, NSC and C with transplant altitude was significantly greater in the low-altitude source of larch than in spruce, suggesting that larch coordinates resource allocation across altitudes through flexible adjustments of carbon and nitrogen metabolism, whereas spruce tends to maintain a relatively stable carbon-nitrogen metabolic state—a conservative strategy that helps reduce the risks associated with cross-altitude transplantation [20,36].

The response patterns of the high-altitude seedling sources differed markedly from those of the low-altitude sources. For the high-altitude source of larch, the sensitivity of instantaneous photosynthetic parameters to transplant altitude change decreased, and no significant differences were observed among transplant altitudes. In contrast, Pn and WUE of the high-altitude source of spruce increased continuously with transplant altitude and reached their optimum at the native altitude, indicating that the high-altitude seedling sources of both species possess strong local adaptation, but the underlying adaptive mechanisms are species-specific.

Such interspecific divergence in responses confirms that the two tree species have developed distinct altitudinal adaptation pathways during growth. Larch exhibited stronger phenotypic plasticity and tended to exploit environmental resources for growth, whereas spruce reduced survival risks by maintaining functional stability, thus supporting Hypothesis (1).

### 4.2. Comparison of Phenotypic Plasticity in Functional Traits Between Tree Species

Studies have shown that under environmental change, larch invests less in leaf construction per unit area and does not need to consume large amounts of resources to maintain organ structures; consequently, larch exhibits higher physiological plasticity in photosynthetic and carbon metabolism traits [37,38]. In contrast, spruce, as an evergreen species, has a long leaf lifespan and a stable leaf structure, requiring greater resource investment for leaf construction and maintenance. Such inherently stable and conservative leaf characteristics restrict the flexible adjustment of various functional traits, resulting in lower overall phenotypic plasticity than larch [21,39].

Larch from low-altitude seedling sources primarily buffers the environmental stress brought by transplantation by regulating nutrient and carbon allocation pathways, which facilitates rapid adaptation within the limited growing season [40]. Notably, under high-altitude seedling source conditions, spruce displayed higher PI values for NSC and N than larch, suggesting that when its needle structure is conservative and difficult to adjust markedly, it may rely more on dynamic carbohydrate regulation to cope with environmental change. The plasticity of leaf SL, SW and SLA was low for both species under different seedling source conditions, indicating that needle morphology possesses strong stability and is under pronounced genetic control. This relative morphological conservatism is a result of long-term environmental selection in conifers and is consistent with the globally observed pattern that trait variation in conifers is generally smaller than in broadleaved species [41,42]. Such differences in adaptive capacity and plasticity among seedling sources provide strong support for the verification of Hypothesis (2).

### 4.3. Associations Among Different Functional Traits of the Tree Species

Pearson correlation analysis showed that several photosynthetic and morphological traits in larch tended to covary, a pattern broadly consistent with the expectations of the global Leaf Economics Spectrum for “resource-acquisitive” strategies: larch seedlings tend to preferentially allocate biomass to leaf area expansion rather than leaf structural construction, and maximize light interception by increasing branch and leaf length, rather than retaining physiological functional substances such as metabolites, reflecting a resource allocation trade-off between “structural growth and physiological metabolism” [43]. The decoupling of the nutrient and carbon metabolism module from the morphological growth module enables seedlings to rapidly adjust morphological structures in response to altitudinal environmental changes without altering the overall metabolic pattern, thereby achieving rapid habitat adaptation within the short growing season—a manifestation of the characteristics of the deciduous “resource-acquisitive” functional type [44].

In contrast to larch, the trait correlation network of spruce exhibited typical characteristics of a conservative resource-use strategy. Most of the morphological parameters, nutrient and carbon allocation traits, and photosynthetic parameters were positively correlated with one another, indicating that at high altitudes, spruce copes with low-temperature stress by simultaneously increasing crown projected area, branch length, non-structural carbohydrate reserves and photosynthetic capacity. Notably, WUE was significantly positively correlated with Protein, but only weakly positively correlated with DBA. This finding suggests that in high-altitude low-temperature environments, increased soluble protein content helps maintain cellular osmotic potential, thereby improving water use efficiency, and that such adaptation comes at the cost of reduced stem diameter; thinner stems can shorten the water transport pathway length and reduce the risk of embolism [45,46]. Leaf N content was negatively correlated with most traits, possibly because under nutrient-limited high-altitude conditions, nitrogen is preferentially allocated to nutrient reserves rather than morphological growth [47].

In summary, larch responds rapidly to environmental changes by coordinating morphological adjustments and nutrient and carbon metabolism. In contrast, spruce adopts a more conservative strategy, exhibiting lower morphological plasticity under high-altitude conditions to ensure survival under prolonged stress, thus supporting Hypothesis (3).

### 4.4. Divergent Regulatory Pathways of Functional Traits Under Different Seedling Source Conditions

For larch, the functional traits showed pronounced differences between seedling source conditions. In the high-altitude seedling source, larch may cope with low-temperature stress by regulating protein synthesis and enhancing structural stability through adjustments in basal diameter (DBA) [48]. In the low-altitude seedling source, the results suggest that, under the relatively mild low-altitude environment, larch tends to optimize resource allocation by adjusting non-structural carbohydrate (NSC) accumulation and protein metabolism to support rapid growth [49,50]. In contrast, the high-altitude seedling source of spruce primarily maintained water balance by regulating transpiration rate (E), reduced leaf construction cost and water loss through lower SLA, and simultaneously optimized carbon and protein metabolism to sustain photosynthetic efficiency; studies indicate that plants tend to maintain high photosynthetic capacity to cope with the short growing season, while enhancing tolerance to cold and intense radiation through morphological traits such as low SLA [51]. In the low-altitude seedling source, spruce may improve light-use efficiency by adjusting the light saturation point (LSP) and adapt to light conditions through adjustments in SLA, while maintaining stable carbon metabolism [52,53]. This reflects the evergreen habit of spruce, characterized by a long leaf lifespan and a stable structure, which predisposes it to buffer environmental stress mainly through enhanced photosynthetic efficiency and leaf morphological adjustments.

The two conifer species adopted divergent regulatory modes in response to altitudinal gradient stress. As a resource-acquisitive species, larch tends to alter its internal substance content to adjust external morphology, thereby achieving rapid adaptation to environmental changes. In contrast, spruce, as a resource-conservative species, exhibited coordinated changes in light response parameters, instantaneous photosynthetic traits, morphological traits and nutrient traits. We suggest that, compared with single-trait adjustment, such coordinated multi-trait changes may represent the key mechanism by which spruce responds to environmental change and maintains its overall functional stability. This reflects a fundamental difference in strategies between the two species, further corroborates the long-term influence of the native altitudinal environment on plant plastic responses, and provides additional support for Hypothesis (3).

## 5. Conclusions

In this study, we used spruce and larch seedlings to systematically compare the altitudinal response differences in four categories of functional traits-light response, instantaneous photosynthesis, leaf morphology and nutrient storage—through a cross-altitude transplant experiment. The results showed that:

(1) Tree functional type was the core factor determining the altitudinal response pattern of seedlings. Larch exhibited higher sensitivity to altitude change, and its photosynthetic strategy traits (Amax, AQE, LSP, E) and morphological strategy traits (PA, SL, SW, SLA) changed significantly with altitude.
(2) The regulation of phenotypic plasticity by seedling source altitude showed a significant functional-type dependence. Larch was regulated by seedling source altitude, with low-altitude seedling sources displaying higher plasticity in traits such as H:D ratio, Tr and NSC. Spruce showed smaller differences between seedling sources, yet exhibited stronger environmental dynamic plasticity than larch in carbon storage and utilization strategies (Sugars, NSC).
(3) The two tree species coped with altitudinal gradient changes through differentiated trait variation pathways. Larch was primarily driven by carbon metabolism and protein synthesis (C, Protein), which was manifested in adjustments of photosynthetic capacity (Pn) and morphology (leaves and branches), thereby achieving rapid adaptation. In spruce, functional traits such as Pn, SLA and NSC were highly coupled; regardless of seedling source, photosynthetic and leaf morphological traits served as the core drivers, and coordinated changes in multiple traits were relied upon to maintain physiological homeostasis. Based on these results, for forest restoration under climate change: low-elevation larch provenances may be given priority in restoration planning due to their higher plasticity in key traits, while spruce, with more stable provenance performance and flexible carbon storage strategies, can serve as a reliable choice across a wider range of elevations. This study was conducted within the altitude range of 1600–2400 m. The adaptability of *Larix principis-rupprechtii* and *Picea asperata* seedlings at other altitude gradients and the changes in their growth strategies after longer-term environmental conditioning require further research.

## Figures and Tables

**Table 1:** In the Table 1, Amax, maximum net photosynthetic rate; LSP, light saturation point; LCP, light compensation point; AQE, apparent quantum efficiency; E, transpiration rate; Pn, net photosynthetic rate; WUE, water use efficiency; SL, leaf length; SW, leaf width; PA, leaf area; LWRatio, leaf length-width ratio; SLA, specific leaf area; BL, new branch length; DBA, branch diameter; H:D ratio, BL:DBA ratio; Protein, leaf protein content; Sugars, leaf soluble sugar content; NSC, leaf non-structural carbohydrate content; C, leaf carbon content; N, leaf nitrogen content.

**Factor**	**Statistic**	**Amax**	**LSP**	**LCP**	**AQE**	**E**	**Pn**	**WUE**	**SL**	**SW**	**PA**
Tree species	*p*-value	<0.001	<0.001	<0.001	0.29	<0.001	<0.001	<0.001	<0.001	<0.001	<0.001
Native elevation	*p*-value	<0.001	<0.05	<0.001	<0.001	0.176	<0.001	<0.001	<0.001	<0.001	<0.001
Transplantation elevation	*p*-value	0.184	0.221	<0.05	0.119	0.145	<0.001	<0.001	<0.001	0.066	<0.001
Tree species × Native elevation	*p*-value	0.072	<0.05	0.39	0.898	0.312	0.396	<0.01	<0.001	0.165	<0.001
Tree species × Transplantation elevation	*p*-value	<0.01	0.479	<0.05	0.117	<0.01	0.679	<0.01	0.509	0.797	0.783
Native elevation × Transplantation elevation	*p*-value	0.346	0.206	0.422	0.967	<0.05	0.605	0.976	<0.001	0.136	0.179
Tree species × Native elevation × Transplantation elevation	*p*-value	<0.001	0.621	0.175	<0.01	<0.05	<0.01	<0.05	0.719	0.404	0.087
**Factor**	**Statistic**	**LWRatio**	**SLA**	**BL**	**DBA**	**H:D ratio**	**Protein**	**Sugars**	**NSC**	**C**	**N**
Tree species	*p*-value	<0.001	<0.001	<0.001	<0.001	<0.001	<0.001	<0.001	0.108	<0.001	<0.001
Native elevation	*p*-value	0.071	<0.01	<0.001	0.63	<0.01	<0.01	0.113	0.562	0.179	<0.001
Transplantation elevation	*p*-value	<0.001	<0.01	0.192	0.836	<0.05	<0.05	<0.001	0.101	<0.001	<0.001
Tree species × Native elevation	*p*-value	0.387	0.467	<0.001	0.07	<0.05	0.162	0.855	0.069	0.254	<0.001
Tree species × Transplantation elevation	*p*-value	0.888	<0.001	<0.05	<0.01	<0.05	<0.001	0.199	0.053	0.179	<0.01
Native elevation × Transplantation elevation	*p*-value	<0.001	0.391	<0.001	0.952	0.896	<0.01	<0.01	0.399	0.275	0.761
Tree species × Native elevation × Transplantation elevation	*p*-value	<0.001	0.877	<0.001	0.594	0.355	<0.001	<0.001	<0.05	0.167	0.566

## Data Availability

The raw data supporting the conclusions of this article will be made available by the authors on request.
